# Comparative Studies on Acetylcholinesterase Characteristics between the Aphids, *Sitobion avenae* and *Rhopalosiphum padi*

**DOI:** 10.1673/031.013.0901

**Published:** 2013-01-31

**Authors:** Y. H. Lu, Y. P. He, X. W. Gao

**Affiliations:** Department of Entomology, China Agricultural University, Beijing 100193, China

**Keywords:** enzyme purification, wheat aphids, acetylcholinesterase sensitivity, inhibitors

## Abstract

The aphids *Sitobion avenae* (Fabricius) and *Rhopalosiphum padi* (Linnaeus) (Hemiptera: Aphidiae) are serious pests on grain crops and usually coexist on late period of wheat growth in China. Bioassays showed that *R. padi* was more susceptible than *S. avenae* to pirimicarb that is used for wheat aphid control, and the determination of acetylcholinesterase (AChE, EC 3.1.1.7) sensitivity showed that the sensitivity of AChE to pirimicarb was significantly higher in *R. padi* than in *S. avenae* (Lu and Gao 2009). AChE is the target enzyme of the carbamates, including pirimicarb, hence, to understand the mechanism responsible for the tolerance difference to carbamate insecticides of *S. avenae* and *R. padi*, we purified AChE from both aphid species using procainamide affinity column and characterized the AChE. The purification factor and yield from *S. avenae* (234.7-fold and 92.9%) were far higher than that from *R. padi* 17.3-fold and 13.9%. The results of substrate and inhibitor specificities of purified enzyme from both *S. avenae* and *R. padi* indicated that the purified enzyme was a typical AChE. The crude AChE extract from *S. avenae* was 5.4-, 4.3- and 8.1-fold less sensitive to inhibition by pirimicarb, methomyl and thiodicarb, respectively, than that from *R. padi*, whereas for the purified AChE, *S. avenae* was only 1.6-, 1.3- and 1.7-fold less sensitive to inhibition by pirimicarb, methomyl and thiodicarb, respectively, than *R. padi*. This suggests that eserine and BW284C51 may bind with other proteins, such as carboxylesterase, in the crude extract to reduce their inhibition against AChE. These results are useful for planning the chemical control of aphids on wheat.

## Introduction

The aphids, *Sitobion avenae* (Fabricius) and *Rhopalosiphum padi* (Linnaeus) are serious pests on wheat in China. Usually, *S. avenae* coexists with *R. padi* in late spring causing damage by direct feeding and as vectors of plant pathogenic viruses. Presently, the control of *S. avenae* and *R. padi* is primarily dependent on the application of insecticides. But the resistance of both aphid species developed slowly so few sprays are currently needed for the control of both *S. avenae* and *R. padi* (Chen et al. 2007). Organophosphate and carbamate insecticides that are antiacetylcholinesterase (AChE) agents are widely used for insect control. AChE hydrolyses the neurotransmitter, acetylcholine, and is, therefore, an important inhibitor at cholinergic synapses in the insect central nervous system (Toutant 1989). AChE is the target for organophosphate and carbamate compounds, which can reduce its sensitivity to such inhibition. Some studies have been done on purified AChE in aphid species, such as greenbug (*Schizaphis graminum*) (Brestkin et al. 1985; Gao and Zhu 2001); cotton aphid (*Aphis gossypii*) (Li and Han 2002). Compared with purified AChE, crude extracts include many contaminants, such as carboxylesterases, that affect the characterization of AChE (Li and Han, 2002).

In previous studies, bioassays showed that *S. avenae* has higher tolerance than *R. padi* to carbamate insecticides (Lu et al. 2009). Since AChE is the target enzyme of carbamate insecticides, its biochemical properties were investigated in *S. avenae* and *R. padi*.

## Materials and Methods

### Insects

Both *S. avenae* and *R. padi* were collected from the same wheat field of Agricultural Experiment Station, China Agricultural University, which has been maintained in the absence of insecticide exposure since May, 2005. These aphids were raised on wheat seedlings (Lu and Gao 2007). Apterous adults were collected in 1.5 mL microcentrifuge tubes, and immediately stored at -80°C.

### Chemicals

Pirimicarb (95% a.i.) was obtained from Wuxi Ruize Chemical Co. Ltd (chinaruize.company.lookchem.cn/), China, Methomyl (90% a.i.) from Jiangsu Changlong Chemical Co. Ltd (www.jschanglong.com/), China, and Thiodicarb (93% a.i.) from SUNDAT(S) PTE LTD (www.37b25a80130.inn.cn/). Acetylthiocholine iodide (ATCh), 1,5-bis(4-allyldimethylammonium phenyl)-pentan-3-one dibromide (BW284C51), eserine hemisulfate salt, acetyl-(β-methyl) thiocholine (MeTCh) and leupeptin were purchased from Sigma Chemical Company (www.sigmaaldrich.com), propionylthiocholine iodide (PrTCh) and butyrylthiocholine iodide (BuTCh), from Aldrich Chemical Company (www.sigmaaldrich.com). 5,5 -dithio-bis-2-nitrobenzoic acid (DTNB) was purchased from Fluka Chemical Company (www.sigmaaldrich.com). Sephadex G-25, tetraethylammonium iodide (Net4I), phenylmethanesulfonyl fluoride (PMSF), procamamide were purchased from Amersham Pharmacia (www.gelifesciences.com). Bovine serum albumin (BSA), ethylenediaminetetraacetic acid (EDTA-Na2) were from Beijing Tongzheng Biological Company (www.bftzbio.com/) and TritonX-100 was from Sigma.

### Purification of AChE

Purification of AChE from both aphid species was performed by affinity chromatography using procainamide as the affinity ligand. The procedure used in this study was according to the method of Gao and Zhu (2001) with some modifications, as follows:

Step 1: Preparation of crude extract. About 1.0 g apterous adults were homogenized in 10 mL ice-cold 0.1 M phosphate buffer (pH 7.5), containing 0.2 M NaCl and 0.5 % (v/v) Triton X-100 and three protease inhibitors (1 mM EDTA-Na_2_, 1 µM leupeptin, and 10 µM PMSF). The homogenate was filtered through cheesecloth and then centrifuged at 41,000 g, 4°C for 1 h. The supernatant, after being filtered through glass wool, was used as crude extract.

Step 2: Chromatography on sephadex G-25. The supernatant (about 9.7 ml for *R. padi* and 9.4 ml for *S. avenae*) from step1 was applied to the sephadex G-25 column (1.6×36 cm) equilibrated with homogenization buffer. The fractions (5 ml each) were collected at the flow rate of 45 mL/h at 4°C. All the fractions containing the AChE activity were collected as the enzyme resource for the next step.

Step 3: Procainamide affinity chromatography and removal of procainamide and condensation. The procainamide-based AChE affinity column was made according to the instructions from the manufacturer. The collected AChE sample from the sephadex G-25 column was loaded on the procainamide-based Sepharose 4B affinity column (1.0×5.5 cm) equilibrated with homogenization buffer. The affinity column was eluted with about 50 mL 0.1 M phosphate buffer (pH 7.5, containing 0.2 M NaCl) at a flow rate of 16 mL/h and the AChE was eluted with 15 mL 0.05 M Net_4_I in 0.1 M phosphate buffer (pH 7.5, containing 0.5 M NaCl) and the fractions of high enzyme activity were pooled together. The collected AChE dialyzed against 0.05 M, pH 7.5 phosphate buffer three times for 3 h. The enzyme was stored at 4°C and used as the enzyme source.

### Substrate specificity and kinetic analysis of crude extract and purified AChE

AChE activities were determined according to the method of Ellman et al. (1961) modified by Gao (1987), using ATCh, PrTCh, MeTCh and BuTCh as substrates. The AChE activities were determined at 6 substrate concentrations from 0.15625 to 5 mM based on the reaction at 30°C for 15 min and the reaction was stopped by the addition of 900 µL, DTNB (0.125 mM) with 40% ethanol, then the optical density (OD) was measured at 412 nm by spectrophotometer (Lambda Bio 40).

### Inhibitor specificity of crude extract and purified AChE, and AChE inhibition by carbamate insecticides

For the inhibitor specificity of AChE test, AChE was preincubated with five different concentrations of each of two inhibitors (eserine, BW284C51) for 5 min at 30°C and then the remaining AChE activity was determined by the addition of substrate according to the above method. The value of median inhibition concentration (I_50_) for the inhibitors were calculated based on log (inhibitor concentration) vs. probit (percentage of inhibition) linear regression.

### Determination of protein concentration

Protein contents of the enzyme preparations were measured according to the method of Bradford (1976) using BSA as standard. The determination was performed at 595 nm with spectrophotometer (Lambda Bio 40).

### Data analysis

All statistical tests were performed by using SAS computer program (USA). Values of Michalelis-Menten constants (*K*_m_) and maximal velocities (*V*_max_) for four substrates were calculated by Enzfitter software (Biosoft, http://www.biosoft.com/).

## Results

### Purification of AChE from *S. avenae* and *R. padi*

**Figure 1.**
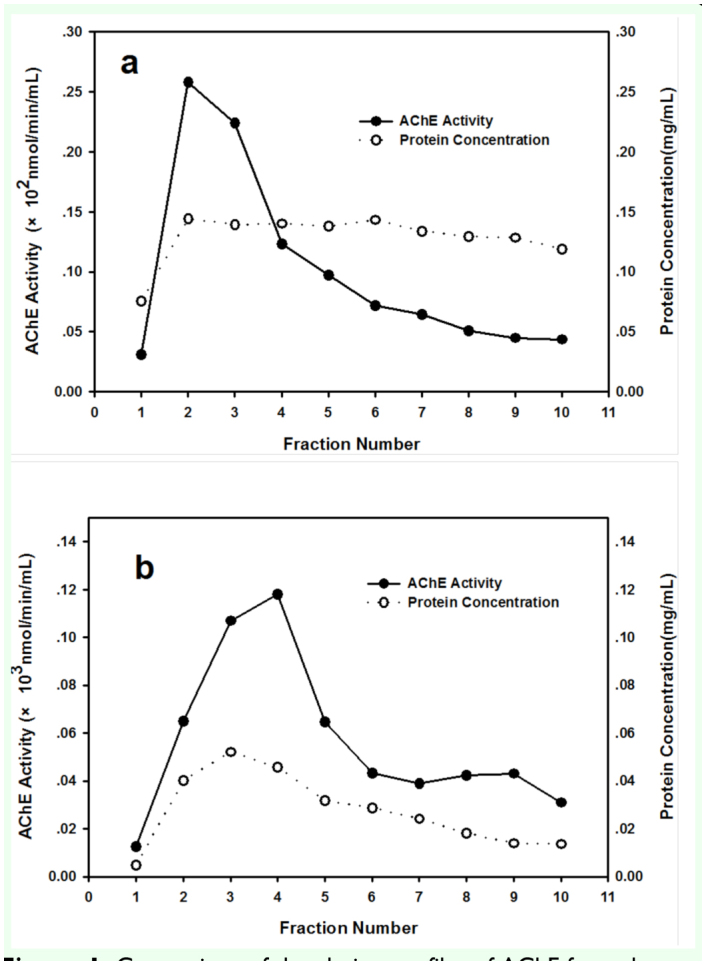
Comparison of the elution profiles of AChE from the procainamide affinity column (1.0 × 5.5 cm) between *Rhopalosiphum padi* (a) and *Sitobion avenae* (b). Flow rate was 16 mL/h, and 1.0 mL fractions except fraction 1, which was 5.0 mL, were collected after the elution buffer containing 0.05 M tetraethylammonium iodide was applied. High quality figures are available online.

AChEs were purified from both *S. avenae* and *R. padi* by procanamide affinity chromatography. The shape of the elution curve for the AChE purification showed no obvious difference between *S. avenae* and *R. padi* (Figure 1). But the purification factor and yield were far higher for *S. avenae* than for *R. padi*. The overall purification factors and yields were 234.7-fold and 92.9 % for *S. avenae*, and 17.3-fold and 13.85 % for *R. padi* respectively (Table 1). The specific activity of AChE after purification by affinity column was 277.00 nmol/min/mg for *S. avenae*, significantly higher than in *R. padi* with the specific activity of AChE with 25.80 nmol/min/mg protein.

### Substrate specificity and kinetic analysis of AChE from *S. avenae* and *R. padi*

**Figure 2.**
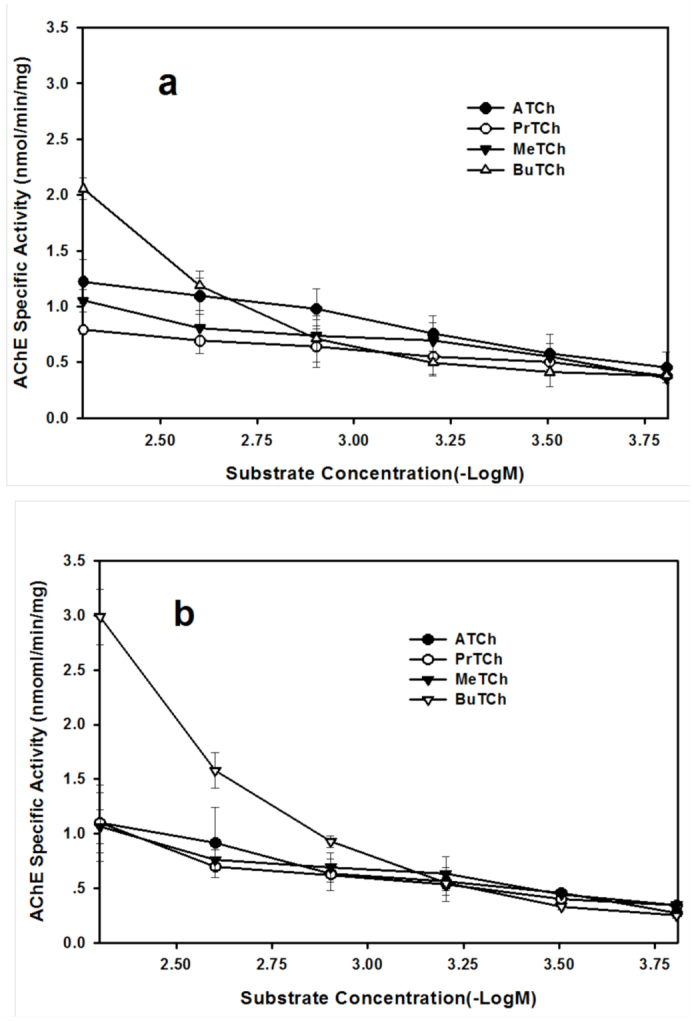
Effect of substrate concentration on crude extract AChE activity from *Rhopalosiphum padi* (a) and *Sitobion avenae* (b). Substrates are ATCh, PrTCh, MeTCh and BuTCh. Each point represents the mean of three replicate assays. Vertical bars indicate standard error of the mean. High quality figures are available online.

**Figure 3.**
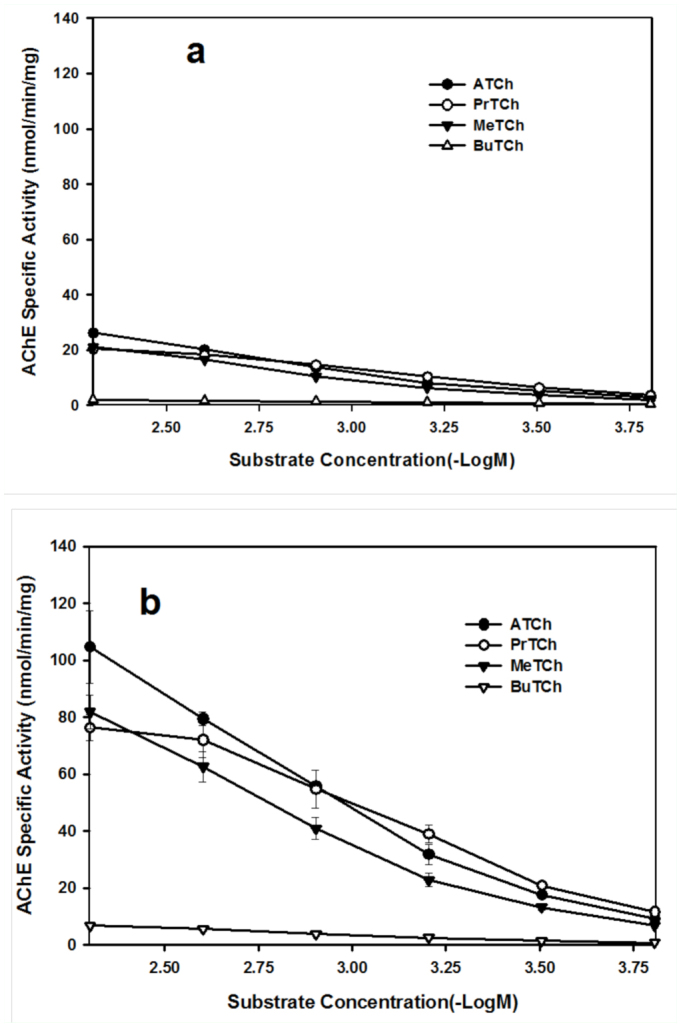
Effect of substrate concentration on purified AChE activity from *Rhopalosiphum padi* (a) and *Sitobion avenae* (b). Substrates are ATCh, PrTCh, MeTCh and BuTCh. Each point represents the mean of three replicate assays. Vertical bars indicate error of the mean. High quality figures are available online.

The effect of substrate concentration on AChE activity using four model substrates, including ATCh, PrTCh, MeTCh and BuTCh, is shown in Figure 2 for the crude extract and Figure 3 for the purified AChE. There were very similar curves hydrolyzing substrates by the crude extract or the purified AChE between *S. avenae* and *R. padi*. There was no significant substrate inhibition at substrate concentration <5 mM for the crude extract or the purified AChE, but substrate inhibition was observed for purified AChE at substrate concentration >10 mM for ATCh and MeTCh. The specific activities of purified AChE from *S. avenae* and *R. padi* at 15 mM of substrates, were 98.6 nmol/min/mg for ATCh, 70.4 nmol/min/mg for MeTCh, 20.3 nmol/min/mg for ATCh and 17.7 nmol/min/mg for MeTCh.

The *K*_m_ values for the crude extract AChE hydrolyzing ATCh, PrTCh, MeTCh and BuTCh were higher in *S. avenae* than in *R. padi* (Table 2). There were no significant differences in *V*_max_ values for the crude extract AChE hydrolyzing ATCh and MeTCh, but the *V*_max_ values hydrolyzing PrTCh and BuTCh were higher in *S. avenae* than in *R. padi*.

However, there were no significant differences in *K*_m_ values for the purified AChE hydrolyzing ATCh, PrTCh and MeTCh, except BuTCh between *S. avenae* and *R. padi* (Table 3). The *V*_max_ values for the purified AChE were significantly higher in *S. avenae* than in *R. padi* when the four substrates were used.

### Inhibitor specificities of crude extract and purified AChE from *S. avenae* and *R. padi*

Table 4 and Table 5 showed that the purified AChE of both *S. avenae* and *R. padi* was more sensitive to inhibition by eserine and BW284C51 than the crude extract AChE. The inhibitory percentages of eserine and BW284C51 at a concentration of 10 µM for the specific activity of the purified AChE were more than 90% and 80% respectively, but only about 50% for the crude extract AChE at the same concentration. I_50_ (median inhibition concentration) values of eserine and BW284C51 for crude extract AChE of were 4.1- and 2.5-times higher in *S. avenae* than in *R. padi* (Table 4), however, I_50_ values of eserine and BW284C51 for the purified AChE were 0.9- and 0.4-fold lower in *S. avenae* than in *R. padi* respectively (Table 5).

## Discussion

AChE was successfully purified from both *S. avenae* and *R. padi* by procainamide-based Sepharose 4B affinity column, the purification factors was 17.3- and 234.7-fold with yields of 13.9 and 92.9% from *R. padi* and *S. avenae*, respectively. The significant differences of purification factor and yield between *S. avenae* and *R. padi* suggested that the purified AChE from *S. avenae* had higher affinity for procainamide than that from *R. padi*. The binding between AChE and the ligand is determined by the affinity between the catalytic center of AChE and its affinity ligand and the hydrophobic interactions between the hexyl “spacer arm” and AChE (Mossoulié and Bon 1976). Thus, the higher purification factor and yield in *S. avenae* suggested structural differences in AChE within the catalytic center, or near the entrance of the gorge of AChE (Ma et al. 2004).

The purified AChE from both *S. avenae* and *R. padi* was highly sensitive to inhibition by eserine and BW284C51, this inhibitory feature has been used as one of the properties to distinguish AChE from butyrylcholine esterase (BChE) (Chatonnet and Lockridge 1989). At a concentration of 10 µM, eserine and BW284C51 inhibited >90% and 80% of the enzyme activity for the purified AChE, but only inhibited about 50% at the same concentration for the crude extract AChE. This suggests that eserine and BW284C51 may bind with other proteins, such as carboxylesterase, in the crude extract that reduces their inhibition against AChE. The substrate inhibition of enzyme activity at high concentration is a typical characteristic for AChE but not for BChE (Toutant 1989). Previous studies indicated that there was no significant substrate inhibition at substrate concentration <5 mM for these four substrates for crude extract AChE, but for purified AChE, the substrate inhibition was observed when substrate concentration >10 mM for ATCh and MeTCh. A similar result was also observed in the purified AChE of the greenbug (Gao and Zhu 2001). This suggests that the purified enzyme from both *S. avenae* and *R. padi* was a typical AChE in substrate and inhibitor specificities.

The I_50_ values of pirimicarb, methomyl and thiodicarb inhibiting the purified AChE were 1.6-, 1.3- and 1.7-fold higher for & *avenae* than for *R. padi* respectively. But the difference of I_50_ values between *S. avenae* and *R. padi* was larger for the crude extract AChE than for the purified AChE. This result suggested that the crude extract AChE contained some ingredients that affected determination of AChE sensitivity, and the purification procedures eliminated these factors, decreasing the difference of I50 values between *S. avenae* and *R. padi*, which resulted in increasing AChE sensitivity to inhibitors (Li and Han 2002).

The carbamate insecticides, pirimicarb and methomyl are widely used in aphid management programs in China. Recently, the difference in the efficacy of pirimicarb between *S. avenae* and *R. padi* has been observed in wheat fields. The susceptible difference to carbamate insecticides between *S. avenae* and *R. padi* was observed in field controls and the laboratory bioassays. Pirimicarb tolerance was higher for *S. avenae* than for *R. padi* (Lu and Gao 2009). The difference of AChE sensitivity together with the metabolism difference likely contributes to the toxicity difference of carbamate insecticides between *S. avenae* and *R. padi*. These results should be very useful in designing wheat aphid management programs.

**Table 1.**
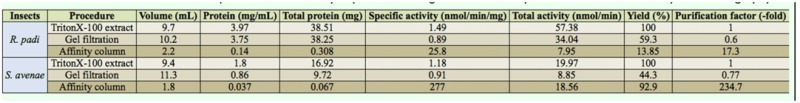
Purification of AChE from *R. padi* and *S. avenae* by sephadex G-25 gel filtration and procainamide-based affinity chromatography.

**Table 2.**
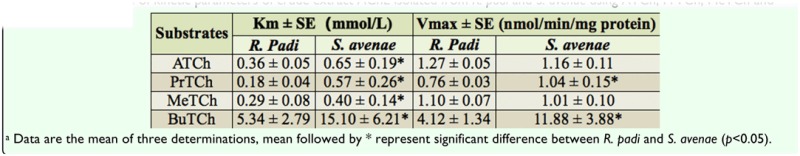
Comparison of kinetic parameters of crude extract AChE isolated from *R. padi* and *S. avenae* using ATCh, PrTCh, MeTCh and BuTCh as substrates^a^.

**Table 3.**
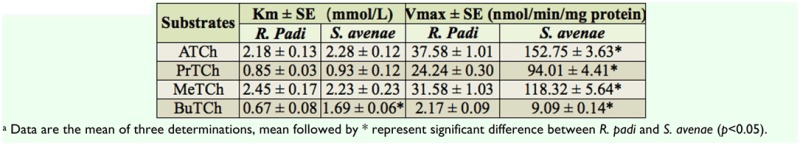
Comparison of kinetic parameters of purified AChE isolated from *R. padi* and *S. avenae* using ATCh,PrTCh, MeTCh and BuTCh as substrates^a^.

**Table 4.**
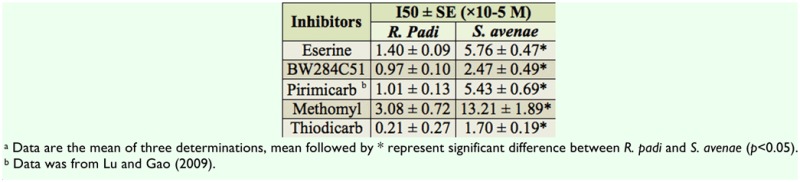
Median inhibition concentration (150) of five compounds in the inhibition of crude extract AChE from *R. padi* and *S. avenae*^a^.

**Table 5.**
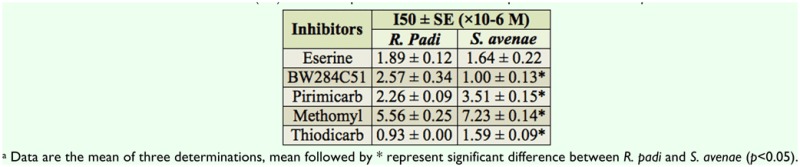
Median inhibition concentration (150) of five compounds in the inhibition of purified AChE from *R. padi* and *S. avenae*^a^.

## Abbreviations

**AChE**,: acetylcholinesterase
**ATCh**,: Acetylthiocholine iodide
**PrTCh**,: propionylthiocholine iodide
**MeTCh**,: acetyl-(β-methyl) thiocholine
**BuTCh**,: butyrylthiocholine iodide
**BW284C51**,: 1,5-bis(4- allyldimethylammonium phenyl)-pentan-3-one dibromide
**DTNB**,: 5,5-dithio-bis-2-nitrobenzoic acid
